# A Novel DNAzyme-Based Fluorescent Biosensor for Detection of RNA-Containing Nipah Henipavirus

**DOI:** 10.3390/bios13020252

**Published:** 2023-02-10

**Authors:** Anastasiia Kirichenko, Ekaterina Bryushkova, Vladimir Dedkov, Anna Dolgova

**Affiliations:** 1 Saint-Petersburg Pasteur Institute, 197101 Saint-Petersburg, Russia; 2 Faculty of Molecular Biology, Moscow State University M.V. Lomonosov, 188512 Moscow, Russia

**Keywords:** DNAzyme 10–23, deoxyribozyme-based biosensor, binary hybridization probe, Nipah virus detection

## Abstract

Nipah virus (NiV) is a zoonotic RNA virus which infects humans and animals in Asian countries. Infection in humans occurs in different forms, from asymptomatic infection to fatal encephalitis, and death occurred in 40–70% of those infected in outbreaks that occurred between 1998 and 2018. Modern diagnostics is carried out by real-time PCR to identify pathogens or by ELISA to detect antibodies. Both technologies are labor-intensive and require the use of expensive stationary equipment. Thus, there is a need to develop alternative simple, fast and accurate test systems for virus detection. The aim of this study was to develop a highly specific and easily standardized system for the detection of Nipah virus RNA. In our work, we have developed a design for a Dz_NiV biosensor based on a split catalytic core of deoxyribozyme 10–23. It was shown that the assembly of active 10–23 DNAzymes occurred only in the presence of synthetic target Nipah virus RNA and that this was accompanied by stable fluorescence signals from the cleaved fluorescent substrates. This process was realized at 37 °C, pH 7.5, and in the presence of magnesium ions, with a 10 nM limit of detection achieved for the synthetic target RNA. Constructed via a simple and easily modifiable process, our biosensor may be used for the detection of other RNA viruses.

## 1. Introduction

Nipah virus (NiV) was detected in 1999 after outbreaks in pigs and humans in Malaysia and Singapore. In Malaysia, 105 of 265 people died and more than 1 million pigs were killed to keep the outbreak under control, which caused serious economic damage [1]. While there have been no more Nipah virus outbreaks in Malaysia or Singapore since 1999, other parts of Asia have reported outbreaks almost annually, especially Bangladesh and India [2]. NiV is an RNA virus belonging to the genus *Henipavirus* of the family *Paramyxoviridae* [3]. The genome of the virus consists of a non-segmented, negative-sense, single-stranded RNA which encodes six structural proteins: nucleocapsid (N), phosphoprotein (P), matrix protein (M), fusion protein (F), glycoprotein (G) and RNA polymerase (L) [4]. The closest relative of the Nipah virus is the Hendra virus (genus *Henipavirus*), with which it has great genetic similarity, a common host and a similar pathogenesis of infection [5]. However, it is important to note that these viruses have different intermediate hosts—for the Hendra virus, horses [6]; and for the Nipah virus, pigs [2]. The natural reservoir for the Nipah virus is the fruit bat (genus *Pteropus*), also known as the flying fox. Flying foxes are endemic to tropical and subtropical regions of Asia, East Africa, the Australian continent and some oceanic islands and have been shown to be associated with NiV outbreaks [7,8]. Infected fruit bats are asymptomatic carriers and can transmit the disease to humans or other animals through close contact or contact with their bodily fluids and feces [9,10]. Nipah virus infection in humans ranges from asymptomatic infection (subclinical) to acute respiratory infection and fatal encephalitis, while death occurred in 40–70% of those infected during documented outbreaks between 1998 and 2018 [11]. The development of an efficient and accurate detection assay for NiV is a task of great importance. Autocatalytic DNA can be used for such an approach.

Deoxyribozymes are short, synthetic, single-stranded DNA oligonucleotides that exhibit catalytic activity. Many of the deoxyribozymes catalyze the chemical cleavage of a phosphodiester bond between nucleotides in the presence of divalent metal ions. Deoxyribozyme 10–23 was chosen as the catalytic core for our test system.

The characteristics of the deoxyribozyme 10–23 (DNAzyme 10–23) make it an attractive tool for use as a sequence-specific endoribonuclease, both in vitro and in vivo [12]. It consists of a catalytic core of 15 nucleotides, surrounded by substrate-binding domains of 6–12 nucleotides each. Substrate-binding domains of the DNAzyme 10–23 must be complementary to nucleotides located both upstream and downstream of the cleavage site.

Any target RNA sequence that is accessible to Watson–Crick pairing and contains a purine–pyrimidine junction can be cleaved by the DNAzyme 10–23 at the phosphodiester located between the purine and pyrimidine residues. The target purine must be unpaired, and all of the flanking nucleotides must be paired. Summarizing the large number of substrate sequences that have been examined to date, there appears to be a preference for R · U compared to R · C sequences at the cleavage site (R = A or G).

It was shown that in the presence of saturating concentrations of substrate, a catalytic activity of the DNAzyme 10–23 increases log-linearly with increasing pH or approximately linearly with increasing concentration of various divalent metal cations (Mn^2+^, Pb^2+^, Mg^2+^, Ca^2+^, Cd^2+^, Sr^2+^, Ba^2+^, Zn^2+^ and Co^2+^, in order of decreasing reactivity), but activity is not affected by monovalent cations. These observations are both connected to a chemical mechanism, involving rate-limiting or metal-assisted deprotonation of the 2′-hydroxyl adjacent to the cleavage site, respectively [13].

**Figure 1:**
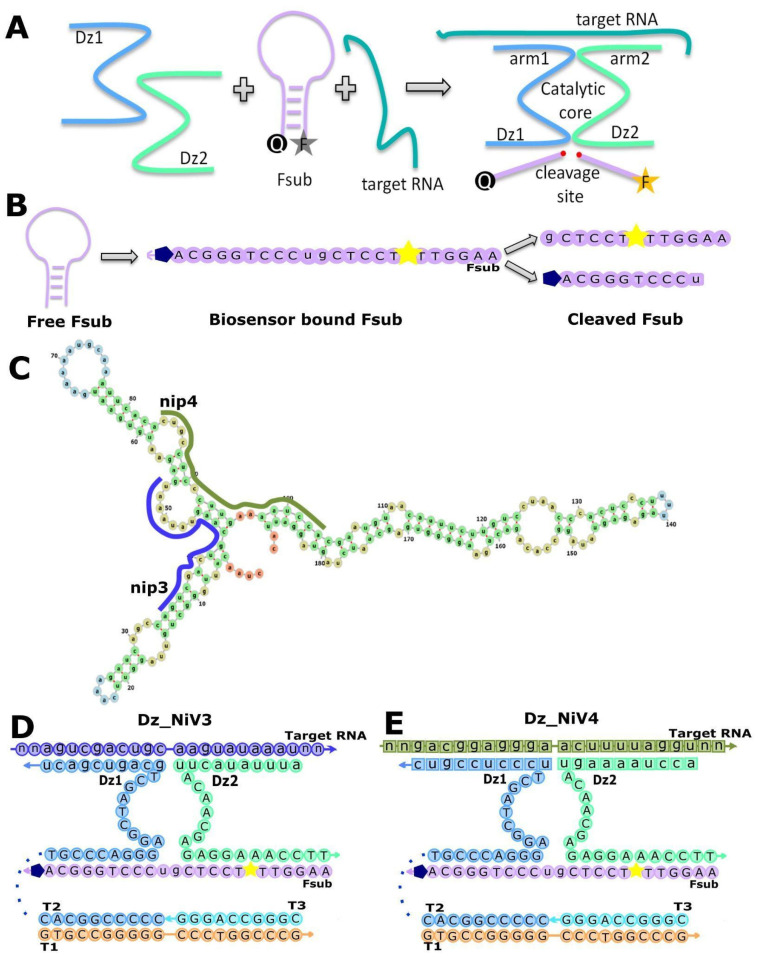
The principle of the deoxyribozyme biosensor design and activity. (**A**) Target RNA detection in the presence of the binary probe and fluorescent substrate (Fsub). (**B**) The Fsub structure before and after its cleavage with Dz_NiV. (**C**) Secondary structure of the G gene of Nipah virus. Purple and green lines indicate RNA regions complementary to DNAzyme arms Dz_NiV3 and Dz_NiV4, respectively. (**D**) Dz_NiV3 design consisting of T1, T3, nip_Dz1_T2_v3 and nip_Dz2_v3. (**E**) Dz_NiV4 design consisting of T1, T3, nip_Dz1_T2_v4 and nip_Dz2_v4. The arrows indicate the 5’-3’ direction of the sequence. The yellow stars indicate the FAM fluorophore, the blue pentagons the BHQ1 quencher and the dotted lines the oligos connecting the hexaethylene glycol linker (HEG).

For detection of the target RNA, two oligonucleotides (binary Dz1 and Dz2) were synthesized; each oligonucleotide consists of a region complementary to the target RNA, half of the catalytic core, and a region complementary to the fluorescent substrate (Figure 1A,B). Thus, if the target RNA is present in solution, the RNA is bound by Watson–Crick base pairing, thereby assembling a catalytic core that cleaves the substrate labeled with a fluorophore and a quencher [14]. Such multicomponent complexes were proposed by Mokany et al. and are called MNAzymes [15].

This research demonstrates the experimental model of the Dz_NiV biosensor based on deoxyribozyme 10–23 for fast and accurate Nipah virus detection. In the future, the creation of a similar biosensor for the closely related Hendra virus is planned. Although there is no specific treatment or vaccine for these viruses, differential diagnosis is important for epidemiological control and understanding how to quickly and efficiently tame an outbreak.

## 2. Materials and Methods

### 2.1. Reagents and Instruments

DNAse/RNAse-free water was purchased from Thermo Fisher Scientific, Inc. (Pittsburgh, PA, USA) and used for all the stock solutions of oligonucleotides and buffers. All oligonucleotides, including fluorescein-labeled RNA (see Table 1 for sequences) were purchased from DNA Synthesis (Moscow, Russian Federation). The oligonucleotides were dissolved in DNAse/RNAse-free water and stored at −20 °C.

The chemicals KCl, MgCl2, urea, NaOH, tetramethylethylenediamine (TEMED), ethylenediaminetetraacetic acid Disodium Salt 2-hydrate (Na2EDTA), ammonium persulfate (APS), Ethidium Bromide solution 1% and Dimethyl Sulfoxide (DMSO) were purchased from PanReacAppliChem (Chicago IL, USA). Agarose, NaCl and tris-(hydroxymethyl)-aminomethane (Tris) were purchased from Helicon (Moscow, Russian Federation). HEPES (4-(2-hydroxyethyl)-1-piperazineethanesulfonic acid) was purchased from Sigma-Aldrich (St. Louis, MO, USA). Acrylamide and N,N’-Methylenebisacrylamide, boric acid, and Triton x100 were purchased from Rosmedbio (St. Petersburg, Russian Federation). Fluorescence measurements were performed using the Axxin T16-ISO Isothermal Fluorescence Reader (Axxin, Australia).

### 2.2. Bioinformatics Analysis

All sequences of NiV available in GenBank (NCBI) were aligned to identify conserved sites using BioEdit [16]. A 169 nt fragment of the G gene (nt positions 9260–9429 in the reference sequence of NiV; GenBank accession number NC_002728.1) was selected as a target for amplification using PLOTCON [17]. The secondary structure of RNA may interfere with the formation of the RNA–biosensor complex. To prevent this, the secondary structures in The Nucleic Acid Package (NUPACK) were predicted [18] and a shorter target region was chosen, taking into account the secondary structure. Short regions (Figure 1C) were selected using BLAST [19].

### 2.3. Experimental Design

A binary-probe design [14] was taken as a basis, and a catalytic core, deoxyribozyme 10–23, was assembled according to complementary base pairing with the target RNA. In both the Dz_NiV 3 and 4 designs, the T1 strand acts as a platform or “core”, and the T2 and T3 strands are complementary to the T1 core, but at the same time the T2 strand is joined to the Dz_1 chain with a hexaethylene glycol linker. The Dz_2 chain is a free oligonucleotide which does not interact with T3 to exclude the possibility of assembling the DNAzyme 10–23 catalytic core in the absence of a target RNA.

The deoxyribozyme becomes active only in the presence of target RNA and magnesium cations; then it can cleave the substrate labeled with the fluorophore and the quencher. The substrate sequence described in [14] was used; it has a hairpin structure to avoid non-specific reactions in the system. The design of the Dz_NiV for each target region is shown in Figure 1D,E.

### 2.4. Assembly of Dz_NiVs

The assembly of the Dz_NiVs was performed by heating a mix of T1–T3 oligonucleotides (10 μM for each strand) for 5 min at +95 °C, followed by cooling to +4 °C, with temperature-decrease increments of 0.5 °C/min. Annealing was carried out in a buffer containing 50 mM HEPES, 50 mM, MgCl2, 20 mMKCl, 120 mMNaCl, 0.03% Triton X-100, 1% DMSO and 0.03% Triton X-100, pH 7.4. The efficiency of the Dz_NiV assembly process was checked by agarose gel electrophoresis in 2% gel prepared in 1x TBE buffer (89 mMTris, 89 mM H3BO3, 2 mM Na2EDTA, pH 8.0). The obtained results were visualized using the gelLITE Gel Documentation System (Cleaver Scientific, Warwickshire, UK).

### 2.5. Cleavage Assay

To prove that substrate cleavage occurred only in the presence of both Dz1 and Dz2, we incubated the assembly and its components together with the fluorescent substrate and Nipah virus RNA for 1 h at 37 °C in the reaction buffer (RB: 50 mM MgCl2, 140 mMNaCl, 5 mMKCl, 50 mM HEPES, 0.25% DMSO, 0.03% Triton X-100, pH 7.4). The concentration of each oligonucleotide was 1 μM. Then, the samples were taken out and put into the denaturing gel-loading buffer (8 M urea in 1x TBE). After that, collected probes were run in the denaturing PAGE (17.5%, 7M urea) at 80 V for 150 min.

### 2.6. Fluorescence Measurements

An oligonucleotide (Fsub) of a loop structure labeled with a fluorophore (FAM) and a quencher (BHQ-1) was chosen to detect a fluorescence signal (495/517 nm). Dz_NiVs were incubated in the presence of short synthetic RNA and Fsub at 37 °C in the reaction buffer (RB) for 20 min. The concentration of all oligonucleotides in the reaction mix, including nip35 and Fsub, was 100 nM. A mixture of reaction buffer, Dz_NiVs and Fsub at the same concentrations was used as a control. During the entire time of incubation, fluorescence was measured using an Axxin T16-ISO Isothermal Fluorescence Reader (Axxin, Australia).

### 2.7. Limits of Detection

The same set of oligonucleotides was incubated for determination of the limits of detection (LODs) for the Dz_NiVs at a 100 nM concentration in the reaction buffer (RB) and different concentrations of short synthetic RNA (5 nM, 10 nM, 20 nM, 50 nM and 100 nM) at 37 °C for 20 min. Incubation and fluorescence measurements were carried out using the Axxin T16-ISO (Axxin, Australia) at a 517 nm wavelength (excitation wavelength: 495 nm). The data for the three independent experiments were plotted using R 4.2.1 [20].

### 2.8. Selectivity Assessment

The selectivity of Dz_NiV was tested in the presence of synthetic RNAs from six other RNA viruses of similar length: Hendra, Machupo, Sabia, Junin, Guanarito and SARS-CoV. Dz_NiV was incubated with RNAs and Fsub at 37 °C in the reaction buffer RB for 1 h; the concentration of all strands was 100 nM, and fluorescence was measured using the Axxin T16-ISO (Axxin, Australia).

## 3. Results

### 3.1. Feasibility Analysis

**Figure 2:**
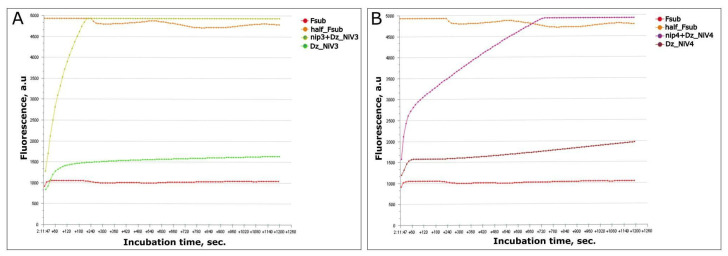
Feasibility analysis of Dz_NiV3 (**A**) and Dz_NiV4 (**B**). The images were directly obtained using the Axxin T16-ISO Isothermal Fluorescence Reader.

In this paper, the Dz_NiV model that was adopted was based on the study of Mokany et al. [15], in which the DNA 10–23 catalytic core was cut into two parts, each of which could bind to a part of the substrate and the target RNA. Based on the results of the bioinformatic analysis, two target RNA sequences were selected, only 35 nucleotides long, which were included in the initially selected G gene fragment (Table 1, nip3 and nip4; Figure 1C). The specificity of these short RNAs for the G gene NiV was further confirmed using the BLAST algorithm [19]. The arms linking the DNAzyme halves with the RNA were 10 nucleotides long each; the target sequence for each DZ_NiV was only 20 nucleotides long. In the presence of the target RNA in solution, hybridization of DNAzymes and RNA occurred, after which we expected to see specific cleavage of the Fsub, labeled FAM and BHQ1, resulting in the release of a short 12-nucleotide fragment (half_Fsub) labeled only with the FAM fluorophore, due to which there was a rapid increase in fluorescence (Figure 2). In order to make sure that the assembly was working, all oligonucleotides were taken at a sufficient concentration of 100 nM.

### 3.2. Selectivity

**Figure 3:**
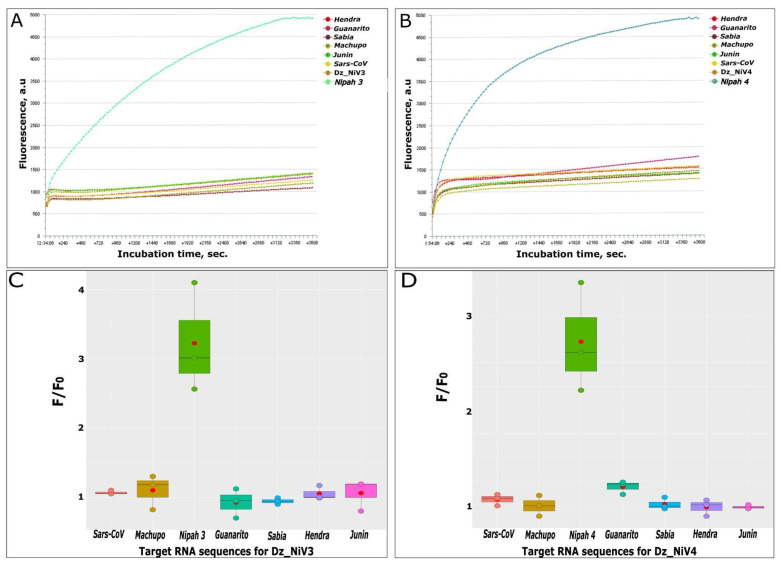
Selectivity of Dz_NiV3 (**A**,**C**) and Dz_NiV4 (**B**,**D**). Curves in the (**A**,**B**) images were directly obtained with the AxxinT16-ISO Isothermal Fluorescence Reader. Images (**C**,**D**) demonstrate fluorescence response from samples containing the different synthetic RNAs. Red dots on the graphs indicate the mean F1/F0 ratios.

The Hendra and Nipah viruses belong to the same genus of Henipaviruses and are the closest to each other in the genus. Their natural host is the flying fox of the genus Pteropus; these bats are also natural reservoirs for SARS-like viruses [5]. To test the specificity of target RNA recognition, Dz_NiVs were incubated with the RNA of six different RNA viruses of similar length: Hendra, Machupo, Sabia, Junin, Guanarito and SARS-CoV viruses. The experiment was repeated three times for each target RNA, and corresponding graphs were built according to the calculated results of these measurements (Figure 3C,D). A positive detection result was only obtained in the presence of Nipah virus RNA in the reaction mixture (Figure 3).

The obtained results are presented as F1/F0 ratios, where F0 means a fluorescence signal from control samples (consisting of the reaction buffer, Dz_NiVs and Fsub at equal concentrations) and F1 means a fluorescence signal from samples, which also included target synthetic RNAs. Our data suggest that, in the presence of the specific target RNA in the measurement sample, the signal/noise ratio (F1/F0) is more than or equal to 1.5.

### 3.3. PH Optimization

**Figure 4:**
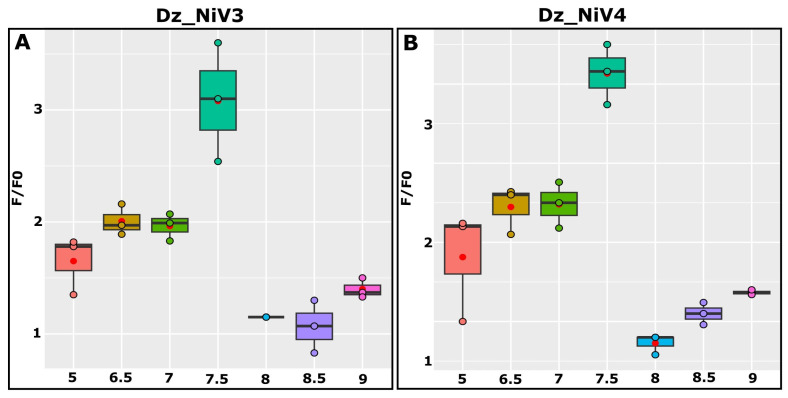
The assessment of pH effects on Dz_NiV3- (**A**) and Dz_NiV4 (**B**)-based biosensor efficiencies. The red dots on the graphs indicate mean values.

The acidity of the reaction buffer has an important effect on the physicochemical properties and biological activities of proteins and nucleic acids. To determine the pH influence on our detection system, the activities of the Dz_NiVs in reaction buffers with pHs = 5, 6.5, 7, 7.5, 8, 8.5 and 9 were tested (Figure 4). The biosensor exhibited the highest activity at a pH equal to 7.5. At a more acidic pH (5–7), the fluorescence signals from both the control and the samples were 4–6 times lower than those obtained at pH 7.5. Although the signal/noise ratio (F1/F0) in this condition was around 2, a too-low value of the registered signal may increase the probability of batch effects. At a pH above 7.5, non-specific denaturation of the fluorescent substrate, leading to a significant decrease in the F1/F0 ratio, was detected (data not shown).

### 3.4. Mg^2+^ Optimization

**Figure 5:**
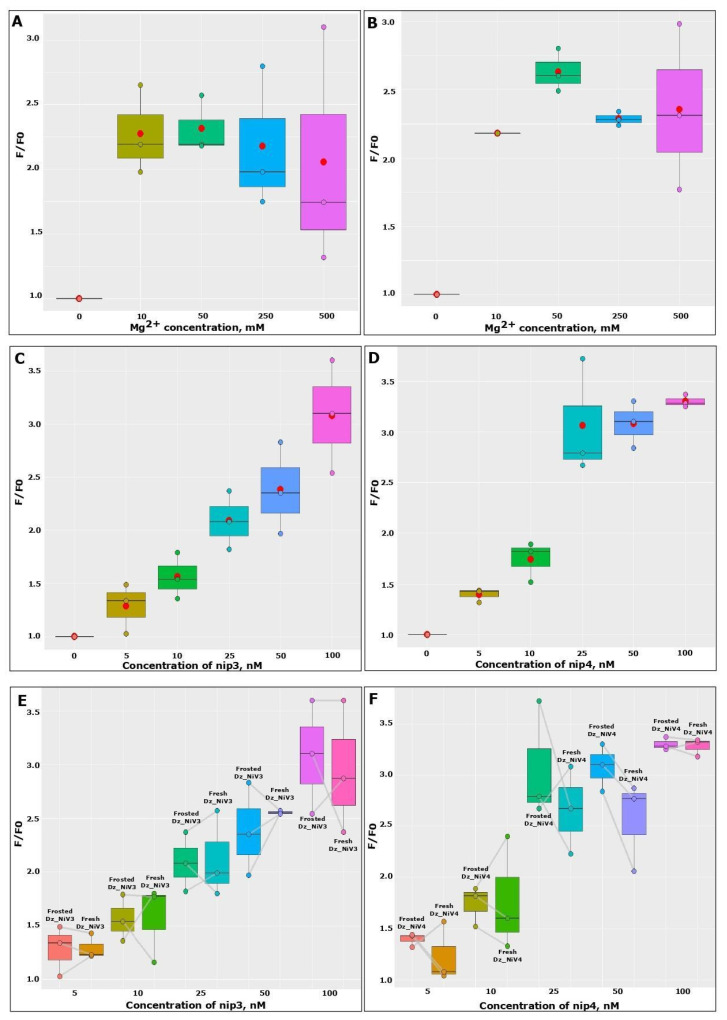
Fluorescence intensity under limiting factors, such as magnesium concentration, for Dz_NiV3 (**A**) and Dz_NiV4 (**B**); target RNA concentrations for Dz_NiV3 (**C**) and Dz_NiV4 (**D**); and triple freezing of Dz_NiV3 (**E**) and Dz_NiV4 (**F**) prior to detection. The red dots on the graphs indicate mean values.

The presence of magnesium cations is necessary for the formation of the correct DNAzyme structure and cutting of the substrate, i.e., the presence of Mg^2+^ ultimately affects the magnitude of the fluorescence signal. To make sure that the result was not random, the analysis was carried out three times, and a graph was drawn based on the results in R. As shown in Figure 5A,B, for both Dz_NiVs, the presence of 50 mM Mg^2+^ was optimal, since with an increase in the magnesium concentration above 50 mM, the catalytic activity decreased and the fluorescence signal values, in samples containing Nipah virus RNA, decreased. The situation was reversed when 10 mM Mg^2+^ was added: the cleavage in the samples with the addition of RNA was active, and the fluorescence values were level with those of the samples containing 50 mM Mg^2+^; however, the fluorescence value of the control also strongly increased, due to which the ratio F/F0 decreased.

### 3.5. Limits of RNA Detection

The next step was to assess the limits of detection (LODs) for the Dz_NiVs. For this experiment, samples containing 100 nM of annealed Dz_NiVs and 100 nM of Fsub were incubated with different amounts of nip3 or nip4 (5 nM, 10 nM, 25 nM, 50 nM and 100 nM) for 20 min at 37 °C. This analysis was also repeated three times. According to the obtained data, the LODs of the Dz_NiVs were 10 nM (Figure 5C,D).

### 3.6. The Effect of Freezing on Dz_NiVs

Due to the fact that the creation of a field test system is planned which could be used in hard-to-reach regions, it is important to understand from the outset that storage and delivery rules may be violated. Therefore, it was decided to evaluate how three freeze–thaw cycles affect the efficiencies of our Dz_NiVs by repeating the test three times. Slight sensitivity drops were found (Figure 5E,F).

## 4. Discussion

Early diagnosis is critical to containing outbreaks of viral diseases and finding the right treatments. Laboratory testing of NiV includes nucleic acid amplification testing, ELISA, immunofluorescence assaying, histopathology and virus isolation and neutralization. Several commercial PCR kits are available, while there is only one commercial source of reagents (not assembled/approved kit) for ELISA testing. These tests require laboratory equipment and qualified personnel, as well as at least 5 h of time to complete the analyses [21].

In the course of this study, a biosensor for the detection of the Nipah virus (Dz_NiV) based on deoxyribozyme 10–23 was developed. It can be easily adapted to different targets; in this study, we selected two targets within the same gene. Signal detection occurs due to cleavage of the hairpin substrate labeled with a fluorophore and a quencher. At present, it can be confidently stated that the sensitivity for each Dz_NiV is 10 nM synthetic RNA variant, which is projected to improve, but the detection time is no more than 20 min and a heating element is not required, as the reaction proceeds at 37 °C. By using several DNAzyme constructs at the same time, an increase in sensitivity and specificity can be achieved.

Fluorescent biosensors typically have a number of disadvantages, such as sensitivity to pH and to the presence of divalent cations in the reaction buffer, the short lifespan of fluorophores, and storage conditions that must be strictly observed. For instance, multiple freeze–thaw cycles should be avoided to maintain the catalytic activity of DNAzymes, and fluorescence-labeled substrates need to be protected from light. Furthermore, unlike calorimetric methods, biosensors need appropriate equipment for the detection of fluorescence signals. In addition, DNAzymes without specific artificial nucleotide modification are susceptible to nuclease activity and may be toxic to cells.

However, the majority of the disadvantages listed are critical only with respect to the usage of DNAzyme biosensors in living systems, such as cell cultures or in vivo models. Regarding diagnostic potential, we may highlight several significant advantages. First of all, the ability to quickly and simply modify the design for the specific recognition of any subtype of the other viruses. In addition, the DNAzyme biosensors have low sensitivity to temperature changes, are easy to use and low-cost, and have high target-sequence selectivity and catalytic activity. They also may be combined into one tube or even one chemical structure for the simultaneous detection of multiple targets, which reduces analysis times and amounts of required materials.

According to our research, currently, there are no articles that present express test systems for the detection of the Nipah virus; however, for some other viruses, test systems have been developed that also use deoxyribozymes for detection. For example, El-Deeb et al. developed a home test system for the detection of SARS-CoV-2, also using DNAzyme 10–23, but which does not involve amplification [22]. The sensitivity limit reached 0.1 fM; however, it takes 3 h to obtain a result and a water bath that maintains a temperature of 55 °C, as well as a Spark fluorescent plate reader, which is unlikely to be found at home.

In the work of Reed et al., a test system for detecting Zika virus using isothermal amplification by the NASBA method in combination with a deoxyribozyme cascade, with a color-change result, was presented; the entire duration of the test takes about 2 h [23]. Due to the amplification and application of DNAzymes, the sensitivity reached ~10^6^ copies/mL viral RNA; this correlates with viral RNA levels of 7 × 10^6^–9 × 10^8^ copies/mL in the blood of symptomatic patients. Among the disadvantages noted by the authors of the article themselves, a thermostat is needed to conduct the test, the NASBA reaction proceeds at 41 °C, and for the deoxyribozymes to work a temperature of 50 °C is required. They also note that, in the current test format, signal generation is suppressed by the NASBA buffer components if more than 10% of the mixture is used.

In the future, the creation of a four-stage detection system is planned. It will consist of sequential reactions, proceed at 37 °C, and include reverse transcription, isothermal amplification (RPA and transcriptions) and direct detection by Dz_NiVs. The convenience of this biosensor model is due to the same catalytic core being used for each target, while the nucleotide binding sequences of the DNAzyme (arms) can be easily modified to suit a panel of fluorescent substrates or target sequences. We are also aiming to develop a biosensor design which will be appropriate for the differential detection of two closely related viruses, Nipah and Hendra, with similar distribution regions and disease symptoms, in one test tube. This can be achieved by creating individual fluorescent substrates for Nipah and for Hendra virus detection, labeled with different fluorophore–quencher pairs. At the same time, the DNAzyme arm sequences will also be modified for complementary recognition of target RNAs. Using an isothermal fluorescence reader with three detection channels (FAM, HEX and ROX), we may detect both Nipah and Hendra virus RNA in parallel at the same time. This test, which can detect co-infection, will be useful because these cases may require different treatment regimens and adjustments to monitor the spread of the viruses.

## 5. Conclusions

In this work, an inexpensive, high-precision, fast biosensor based on deoxyribozyme 10–23 was constructed for the detection of the Nipah virus. In the presence of Nipah virus RNA and magnesium ions, the DNAzyme catalytic core is assembled, which cleaves the hairpin substrate labeled with a fluorophore and a quencher, due to which the fluorophore is released into the solution. To obtain a detection result, 20 min is enough, and the incubation takes place at 37 °C, which is important for analysis in the field. The biosensor requires pre-assembly, but it has been shown that it can be stored at −20 °C and subjected to three freeze–thaw cycles without loss of catalytic activity, which will be important in creating a test system with pre-amplification and detection steps and transferring it to usage. In the future, we plan to optimize the DzNiV biosensor for the detection of Nipah viral RNA in clinical samples. Biological samples may contain insufficient numbers of virus copies for detection by the Dz_Niv biosensor, so the developed test system will include the following steps: reverse transcription, amplification, transcription and detection.

## Figures and Tables

**Table 1:** Sequences of the oligonucleotides used in this study.

Name	Sequence (5′-3′)
T1	GTGCCGGGGGCCCTGGCCCG
T3	CGGGCCAGGG
Fsub	AAG GTTFAMTCC TCg uCC CTG GGC ABHQ1
nip3	aucagccagucgacugcaaguauaaaugagaaugu
nip_Dz1_T2_v3	CCCCCGGCAC/HEG/TGCCCAGGGAGGCTAGCTgcagucgacu
nip_Dz2_v3	auuuauacuuACAACGAGAGGAAACCTT
nip4	auucacacugccucccuugaaaauccacgaaugua
nip_Dz1_T2_v4	CCCCCGGCAC/HEG/TGCCCAGGGAGGCTAGCTagggaggcag
nip_Dz2_v4	uggauuuucaACAACGAGAGGAAACCTT
half_fsub	AAG GTTFAM TCC TCg
G_RNA	gcaaugccuccuucugauaagacagu
S_RNA	cugcugguucgccaauacaaaucuaauaaaau
J_RNA	uagaauagcaccuugacuucucaccuguuuu
Sars-CoV	cuugcacuagcacacacuuugcuuuugcuugugcugacgguacucgacau
MV2_RNA28	agauaaauaaacugaauaacaccugga

FAM—6-fluorescein amidite; BHQ-1—Black Hole Quencher-1; HEG—hexoethylene glycol linker; Dz—deoxyribozyme; Fsub—fluorescent substrate; nip3 and nip4—targeted synthetic RNAs of the Nipah virus; half_fsub—half of the fluorescent substrate (without quencher) released during the cleavage reaction; G_RNA—synthetic RNA of the Guanarito virus; S_RNA—synthetic RNA of the Sabia virus; J_RNA—synthetic RNA of the Junin virus; MV2_RNA28—synthetic RNA of the Machupo virus; uppercase letters indicate DNA nucleotides; lowercase letters indicate RNA nucleotides.

## Data Availability

Not applicable.
